# The Species of *Rhimphoctona* (*Xylophylax*) (Hymenoptera: Ichneumonidae: Campopleginae) Parasitizing Woodborers in China

**DOI:** 10.1673/031.010.0401

**Published:** 2010-02-25

**Authors:** You-Qing Luo, Mao-Ling Sheng

**Affiliations:** ^1^ The Key Laboratory for Silviculture and Conservation of Ministry of Education, Beijing Forestry University, 100083, P. R. China; ^2^ General Station of Forest Pest Management, State Forestry Administration, 58 Huanghe North Street, Shenyang 110034, P. R. China

**Keywords:** Hymenoptera, lchneumonidae, *Rhimphoctona*, *Xylophylax*, new species, woodborer

## Abstract

Four species of *Rhimphoctona* (*Xylophylax*) collected from P. R. China are reported. Two of them are new to science: *Rhimphoctona* (*Xylophylax*) *maculifemoralis* Luo and Sheng, sp.nov. reared from *Tetropium castaneum* (Linnaeus), and *Rhimphoctona* (*Xylophylax*) *immaculata* Luo and Sheng, sp.nov. One is a new record for China, *R*. (*Xylophylax*) *rufocoxalis* (Clément 1924) reared from *T. castaneum* (Linnaeus). The other is *R*. (*Xylophylax*) *lucida* (Clément 1924) reared from *Monochamus saltuarius* Gebier, *Tetropium gabrieli* Weise and *Asemus* sp. A key to species known in China is provided.

## Introduction

*Rhimphoctona* (*Xylophylax*) belonging to subfamily Campopleginae of the lchneumonidae (Hymenoptera) are important parasitoids of woodborers. Based on the most current version of Taxapad (Yu et al. 2005), there are 13 Palearctic, 14 Nearctic, 1 Holarctic, and 1 Oriental species of *Rhimphoctona*. The European species of the genus *Rhimphoctona* Förster were revised by K. Horstmann (1980). The Nearctic species of the subgenus *Xylophylax* Kriechbaumer were reported by M. Sanborne (1986). The status of the genus was elucidated by D. Wahl (1991).

The genus has not been studied thoroughly in the Oriental and Palearctic regions of China. Only one species, *Rhimphoctona* (*Xylophylax*) *lucida* (Clément 1924), has been recorded (Sheng et al. 2002). In the present paper, four species of subgenus *Xylophylax* Kriechbaumer from P. R. China are reported.

The morphological terminology follows Gauld (1997). Wing vein nomenclature follows Mason (1986).

**Figure 1, 2, 3, 4:**
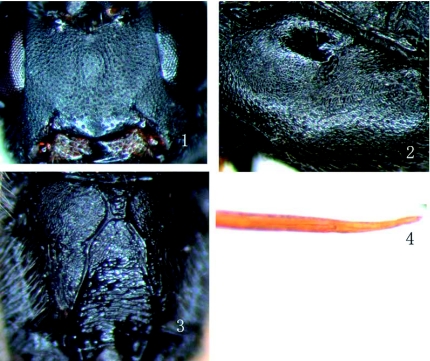
*Rhimphoctona* (*Xylophylax*) *maculifemoralis*, sp.nov. 1. Face. 2. mesopleuron. 3. Propodeum. 4. Ovipositor. High quality figures are available online.

The type specimens of *R*. (*Xylophylax*) *rufocoxalis* (Clément) were lent from Zoologische Staatssammlung München, Munich, Germany, and the specimens of *R*. (*Xylophylax*) *lucida* (Clément) for comparison were from K. Horstmann, Zoologie III, Biozentrum, Wuerzburg, Germany.

## Taxonomy

**Subgenus *Xylophylax* Kriechbaumer 1878 *Xylophylax* Kriechbaumer  1878:210. Type species: *Pyracmon*  (*Parapyracmon*) *rufocoxalis* Clément 1924:125. Designated by Townes (1970).**
**Diagnosis.** Lower tooth generally much longer than upper tooth. Vein 2m-cu of the forewing meeting vein M closer to vein 3r-m than to 2r-m. Metasomal segment 1 short and stout, with large, deep glymma, its spiracle near middle. Ovipositor cylindrical, apical portion upturned. Face of male partly to entirely yellow. Clypeus of female partly to completely reddish brown.

***Rhimphoctona* (*Xylophylax*) *maculifemoralis*, sp.nov.**
**Diagnosis.** Apical portion of antenna somewhat compressed. Hind leg black, its dorso-basal portion of femur with brownish-yellow fleck. Face of male is yellow, with a small black fleck at upper centre.
**Description.** Female. Body length 7.8 to 8.5 mm. Forewing length 6.0 to 6.2 mm. Ovipositor sheath length about 3 mm.
**Head.** Face almost flat, about 1.5 times as wide as long, with dense granulation and distinct punctures, upper margin weakly concave centrally. Clypeus (Figure 1) flat, basal portion with dense punctures. Apical portion smoother, apex with a weak median projection. Mandible strong, with transverse punctures, lower tooth long and acute, 2.0 times as long as upper tooth. Malar space rough, 0.8 times as long as basal width of mandible. Gena coriaceous with very sparse punctures, hind portion weakly expanded, in lateral view 0.8 to 0.9 times as long as width of eye. Vertex evenly convex, nearly the same texture as gena. Interocellar area with median longitudinal concavity. Postero-ocellar line about as long as ocular-ocellar line. Frons concave toward centre, with fine oblique lines and a short median longitudinal carina. Antenna very thin, with 43 to 44 flagellomeres, apical portion somewhat compressed. Occipital carina complete.
**Mesosoma.** Pronotum slightly rough, with irregular oblique wrinkles, upper portion with fine punctures. Without epomia. Mesoscutum evenly convex, finely coriaceous and with very fine punctures. Notaulus very weak, as a vestige on front portion of mesoscutum. Scutellum smooth, with distinct, fine punctures. Mesopleuron (Figure 2) and mesosternum finely coriaceous with very fine punctures and wrinkles below speculum. Speculum small. Metapleuron with indistinct punctures. Submetapleural carina complete and strong. Wing brownish hyaline, 1cu-a opposite or almost opposite 1-m. Areolet a slanting quadrangle, receiving vein 2m-cu at 0.75 to 0.8 distance from vein 2r-m to 3r-m. Vein 2-Cu as long as 2cu-a. Vein 1-cu strongly inclivous, about 3.0 times as long as cu-a. Coxae texture as mesosternum. Hind femur strongly compressed. Claw small, its base pectinate. Propodeum (Figure 3) weakly rough, finely coriaceous. Costula absent. Area petiolaris with transverse wrinkles. Area superomedia separated from area basalis by strong transverse carina and combined with area petiolaris. Spiracle approximately circular (slightly elliptic).
**Metasoma.** Terga finely coriaceous. Hind half of metasoma compressed. First tergum 2.5 to 2.6 times as long as its apical width, slender, only postpetiole slightly wider. Median dorsal carina absent. Dorsolateral carina present, reaching to hind end of glymma. Glymma deep. Spiracle very small, slightly convex. Second tergum elongate, 1.5 times as long as its apical width. Ovipositor sheath very slim, about as long as hind tibia. Ovipositor (Figure 4) without subapical dorsal notch.
**Color.** Black. Mandible yellow to yellowish brown. Maxillary palpus, labial palpus, tegula, front tarsus, second segment of middle trochanters, basal lower portion of middle femur, and middle tibia blackish brown. Front femur and tibia and apical portion of middle femur brown to reddish brown. Basal dorsal portion of hind femur with a yellowish brown fleck. Stigma and veins brownish black.
**Male.** Body length 8.5 to 10.0 mm. Forewing length 6.0 to 6.5 mm. Antenna with 43 to 45 flagellomeres. Face except median black fleck, clypeus, mandible except tooth, malar space, lower portion of gena, maxillary palpus, labial palpus, lower profile of scape, front and middle legs (except basal end of coxae black and tarsi blackish brown) yellowish brown.
**Type material.** Holotype ♀, CHINA: Maixiu Forestry farm, Huangnan, Qinghai Province, 2006.VI.14, L. Liu. Paratype: 31 ♂♂ 9♀♀, same data as holotype, except 2006.VII.4 to VIII.9. All types are deposited in Insect Museum, General Station of Forest Pest Management (GSFPM), State Forestry Administration, Shenyang, P. R. China.
**Host.** All specimens were reared from wood of *Picea crassifolia* Komarov from which many *Tetropium castaneum* (Linnaeus) (Coleoptera: Cerambycidae) emerged.
**Distribution.** China (Qinghai).
**Etymology.** The name of the new species is based on upper-basal portion of hind femur with brownish yellow fleck.
**Remarks.** The new species resembles *R*. (*Xylophylax*) *lucida* (Clément 1924), but can be distinguished from the latter by its ovipositor approximately as long as hind tibia; hind leg, except dorso-basal portion of femur with brownish yellow fleck, black; face of male yellow, with a small black fleck at upper centre.

**Figures 5, 6, 7, 8, 9:**
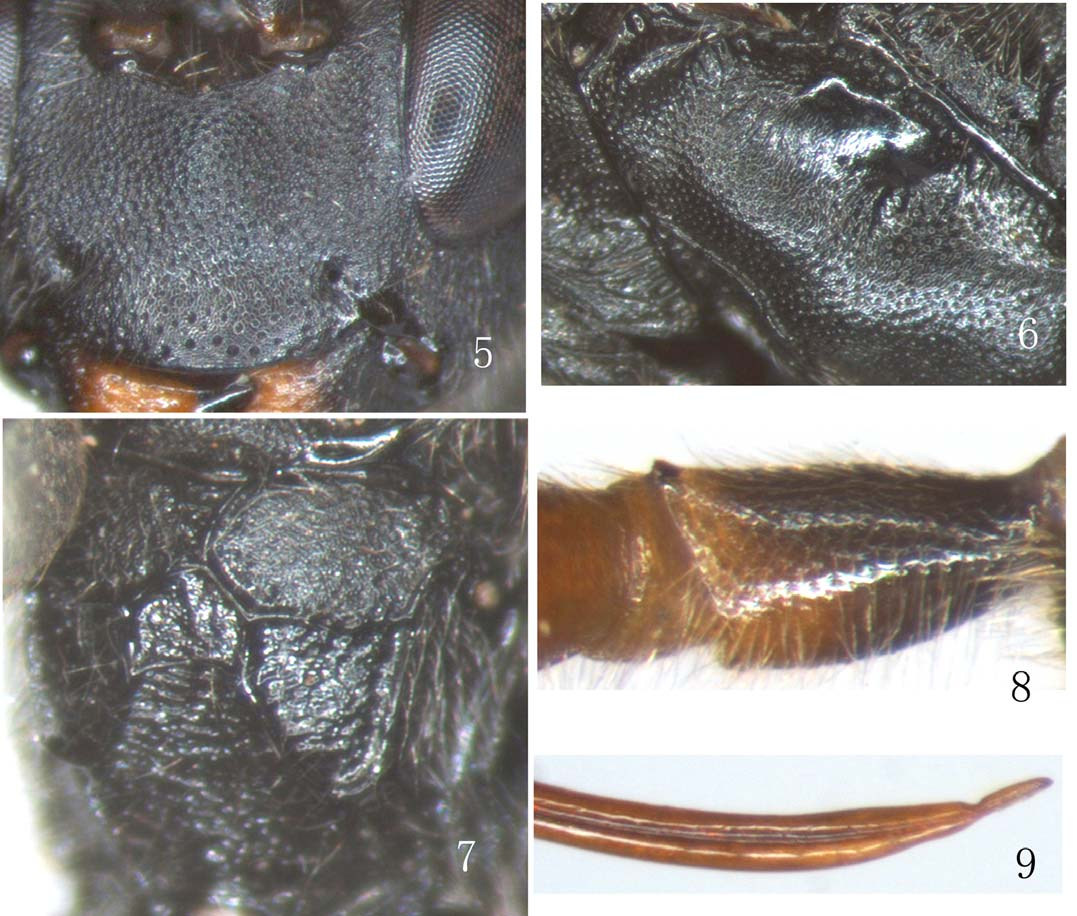
*Rhimphoctona* (*Xylophylax*) *immaculata*, sp.nov. 5. Face. 6. Mesopleuron. 7. Propodeum. 8. First trochanter of front leg. 9. Ovipositor. High quality figures are available online.

***Rhimphoctona* (*Xylophylax*) *immaculata*, sp.nov.**
**Diagnosis.** Apical margin of clypeus obtuse, without median projection. First trochanter of front leg with an apical tooth on front side. Ovipositor with a small subapical dorsal notch. Clypeus and hind legs of female entirely black. Mesosternum of male entirely yellow.
**Description.** Female. Body length about 9.5 mm. Forewing length about 7.5 mm. Ovipositor sheath length about 3 mm.
**Head.** Face 2.0 times as wide as long, with fine dense granulation and punctures, slightly convex centrally, upper margin with a small median protuberance. Clypeus (Figure 5) coriaceous with sparse punctures, median portion slightly concave, apical portion smoother, apical margin obtuse, without projection. Mandible strong, coriaceous with sparse fine punctures, lower tooth long and acute, 1.6 times as long as upper tooth. Malar space slightly concave and rough, 0.65 times as long as basal width of mandible. Gena coriaceous with very sparse fine punctures, in lateral view about 0.83 times as long as width of eye. Vertex evenly convex, nearly the same texture as face, but more sparse punctures. Interocellar area weakly convex, with shallow median concavity. Postero-ocellar line about 0.9 times as long as ocular-ocellar line. Frons evenly concave, the same texture as face, lower median portion with very fine transverse wrinkles. Antenna short, with 28 flagellomeres. Occipital carina complete.
**Mesosoma.** Front portion of pronotum with fine longitudinal wrinkles, lateral concavity with short and dense transverse wrinkles, upper portion with fine punctures. Epomia indistinct. Mesoscutum evenly convex, with even and fine punctures. Notaulus very weak, as a vestige on subanterior portion of mesoscutum. Scutellum evenly convex, smooth, with distinct fine punctures. Postscutellum slightly rough, with dense and fine punctures. Mesopleuron (Figure 6) and mesosternum with even and fine punctures, the latter denser than the former, in front of speculum with fine oblique wrinkles. Speculum smooth, with unclear fine oblique lines. Metapleuron slightly rough, with dense and indistinct punctures. Submetapleural carina complete and strong. Wing brownish hyaline, 1cu-a distad of 1-m. Areolet a slanting quadrangle, receiving vein 2m-cu slightly basad of vein 3r-m. Vein 2-Cu as long as 2cu-a. Vein 1-cu slightly inclivous, about 3 times as long as cu-a. First trochanter (Figure 8) of front leg with an apical tooth on front side. Claw small, distinctly pectinate. Propodeum (Figure 7) weakly rough, unclearly punctured, with strong carina. Costula complete and strong. Area superomedia separated from area basalis by strong transverse carina, and combined with area petiolaris. Area superomedia slightly wider than length, connecting with costula at its middle. Apical portion of area superomedia and area petiolaris with transverse wrinkles. Spiracle approximately circular (slightly elliptical).
**Metasoma.** Apical portion, from third tergum to end of metasoma, strongly compressed. First tergum gradually wider behind, about 2.6 times as long as its apical width, slightly rough, apical portion with very weak longitudinal wrinkles. Median dorsal carina absent. Dorsolateral carina strong before spiracle, the rest weak. Glymma deep. Spiracle small, slightly concave. Second tergum extremely finely coriaceous, approximately as long as its apical width. Ovipositor sheath very slim, about 0.9 times as long as hind tibia. Ovipositor (Figure 9) evenly upturned, with a distinct subapical dorsal notch.
**Color.** Black. Maxillary palpus and labial palpus unevenly blackish brown. Median portion of mandibles, front femur and outer side of middle femur reddish brown. Front and middle tibiae brown, their tarsi brownish black.
**Male.** Body length about 10.5 mm. Forewing length about 7.5 mm. Antenna with 41 flagellomeres. Subposterior portion of gena swollen. Face, clypeus, mandible except tooth, malar space, gena, frontal orbit, maxillary palpus, labial palpus, scape, pedicel, coxae and trochanters of front and middle legs, propleuron, lower portion of mesopleuron, mesosternum and tegula yellow. Front and middle legs and hind trochanter yellowish brown. Coxa except basal end of upper profile and femur of hind leg reddish brown. Hind tibia obscurely reddish brown. Hind tarsus blackish brown.
**Type material.** Holotype♀, CHINA: Baotianman Natural Preserve, Henan Province, 1280m, 2006.V.25., Xiao-Chen Shen. Paratype: 1♂, same data as holotype. All types are deposited in Insect Museum, General Station of Forest Pest Management (GSFPM), State Forestry Administration, Shenyang, P. R. China.
**Distribution.** China (Henan).
**Etymology.** This new species name is based on the color of the female body, entirely black without markings.
**Remarks.** This species is similar to *R*. (*Xylophylax*) *rufocoxalis* (Clément 1924), but can be distinguished from the latter by the following characters: clypeus and hind legs entirely black; area superomedia of propodeum wider than long; costula complete and strong, connecting with areola at the middle of the latter. *R*. (*Xylophylax*) *rufocoxalis* (Clément): clypeus yellow; hind legs, at least coxae, trochanters and femora, reddish brown; area superomedia longer than its width; costula weak and incomplete, connecting with areola behind the middle of the latter.

**Figure 10:**
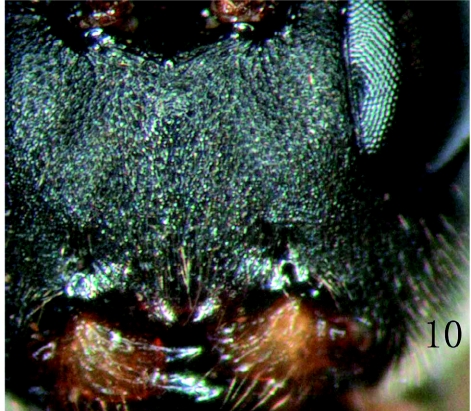
*Rhimphoctona* (*Xylophylax*) lucida (Clément, 1924). Face. High quality figures are available online.

**Figure 11:**
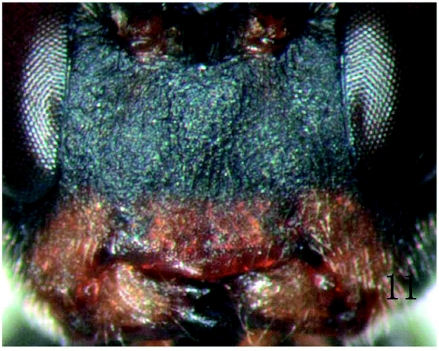
*Rhimphoctona* (*Xylophylax*) *rufocoxalis* (Clément, 1924). Face. High quality figures are available online.

***Rhimphoctona* (*Xylophylax*) *lucida* (Clément 1924)**
*Pyracmon* (*Parapyracmon*) *lucidus* Clément 1924. Deutsche Entomologische Zeitschrift, 1924:130.
Specimens examined: 1 ♀, CHINA: Chuo'er, Inner Mongolia, 1981.VI.18.; 9♀♀3♂♂, CHINA: Tuqian, Heilongjiang Province, 1989.VI.7., M.-L. Sheng, reared from cocoons that were collected from the galleries of *Tetropium gabrieli* Weise and *Asemus* sp. (Coleoptera: Cerambycidae); 3♀♀9♂♂, CHINA: Daxing'gou, Jilin Province, 2005. VI. 17., M.-L. Sheng, reared from cocoons that were collected from the galleries of *Monochamus saltuarius* Gebler (Coleoptera: Cerambycidae).

***Rhimphoctona* (*Xylophylax*) *rufocoxalis* (Clément 1924)**
*Pyracmon* (*Parapyracmon*) *rufocoxalis* Clément 1924. Deutsche Entomologische Zeitschrift, 1924:125.
Lectotype ( ♀ ): “Starnbg. 13.6.58 Krchb.” (München).
  New record for China.
Specimens examined: 1 ♀6♂♂, Maixiu forestry farm, Huangnan, Qinghai province, June 22 to August 4, 2006, Li Liu, reared from wood of *Picea crassifolia* Komarov from which many *Tetropium castaneum* (Linnaeus) (Coleoptera: Cerambycidae) emerged (new host record).

**Key to species of *Rhimphoctona* (*Xylophylax*) known in China**
1. Propodeum with costula absent. Ovipositor sheath about as long as hind tibia. Hind coxa, trochanter and femur black. Basal dorsal portion of hind femur with a yellowish brown fleck *R.* (*Xylophylax*) *maculifemoralis* Luo and Sheng, sp.nov.
Propodeum with distinct costula. 2
2. Clypeus reddish brown. Hind coxa, trochanter and femur red or reddish brown. Costula incomplete *R.* (*Xylophylax*) *rufocoxalis* (Clément)
Clypeus black, or only apical margin slightly brownish centrally. Hind leg with at least coxa black. Costula complete. 3
3. Apical margin of clypeus with a weak median projection. First trochanter of front leg without an apical tooth on front side. Ovipositor sheath about 1.5 times as long as hind tibia. *R.* (*Xylophylax*) *lucida* (Clément)
Apical margin of clypeus without a weak median projection. First trochanter of front leg with an apical tooth on front side. Ovipositor sheath about 0.9 times as long as hind tibia. *R.* (*Xylophylax*) *Immaculata* Luo and Sheng, sp.nov.

## References

[bibr01] Clément E (1924). Opuscula Hymenopterologica I. Die Ophioninen-Gattungen Pyracmon Hlgr. und *Rhimphoctona* Foerst. (lchneumonidae, Ophioninae).. *Deutsche Entomologische Zeitschrift*.

[bibr02] Gauld ID, Wahl D, Bradshaw K, Hanson P, Ward S (1997). The lchneumonidae of Costa Rica, 2. Introduction and keys to species of the smaller subfamilies, Anomaloninae, Ctenopelmatinae, Diplazontinae, Lycorininae, Phrudinae, Tryphoninae (excluding Netelia) and Xoridinae, with an appendices on the Rhyssinae.. *Memoirs of the American Entomological Institute*.

[bibr03] Mason WRM (1986). Standard drawing conventions and definitions for venational and other features of wings of Hymenoptera.. *Proceedings of the Entomological Society of Washington*.

[bibr04] Sheng ML, Kou MJ, Cui YS, Bing JC, Sun SP, Su W (2002). A list of Ichneumonids parasitizing tree borers in North China.. *Journal of Gansu Forestry Science and Technology*.

[bibr05] Horstmann K (1980). Revision der europäischen Arten der Gattung *Rhimphoctona* Förster (Hymenoptera, lchneumonidae).. *Nachrichtenblatt der Bayerischen Entomologen*.

[bibr06] Meyer NF (1935). Parasitica of the family lchneumonidae of the USSR and adjacent countries. Part 4. Ophioninae.. *Leningrad. Akademia Nauk SSSR Press*.

[bibr07] Sanborne M (1986). On the correct application of *Rhimphoctona* Förster (Hymenoptera: lchneumonidae). *Contributions to the American Entomological Institute*.

[bibr08] Sanborne M (1986). The subgenera of *Xylophylax* (Hymenoptera: lchneumonidae) with descriptions of two new eastern Palearctic species.. *Contributions to the American Entomological Institute*.

[bibr09] Sanborne M (1986). Revision of the Nearctic species of *Xylophylax* Kriechbaumer (Hymenoptera: lchneumonidae).. *Contributions to the American Entomological Institute*.

[bibr10] Townes HK (1969). The genera of lchneumonidae, Part 3.. *Memoirs of the American Entomological Institute*.

[bibr11] Wahl DB (1991). The status of *Rhimphoctona*, with special reference to the higher categories within Campopleginae and the relationships of the subfamily (Hymenoptera: lchneumonidae).. *Transactions of the American Entomological Society*.

[bibr12] Yu DS, Horstmann K (1997). A catalogue of world lchneumonidae (Hymenoptera).. *Memoirs of the American Entomological Institute*.

[bibr13] Yu DS, van Achterberg K, Horstmann K (2005). World Ichneumonoidae 2004..

